# A Noninvasive Accurate Measurement of Blood Glucose Levels with Raman Spectroscopy of Blood in Microvessels

**DOI:** 10.3390/molecules24081500

**Published:** 2019-04-17

**Authors:** Nan Li, Hang Zang, Huimin Sun, Xianzhi Jiao, Kangkang Wang, Timon Cheng-Yi Liu, Yaoyong Meng

**Affiliations:** MOE Key Laboratory of Laser Life Science & Laboratory of Photonic Chinese Medicine, College of Biophotonics, South China Normal University, Guangdong 510631, China; 2015021267@m.scnu.edu.cn (N.L.); 18363034332@163.com (H.Z.); sunhuimin@163.com (H.S.); 13503040644@163.com (X.J.); 15625072392@163.com (K.W.); ky201520162zxjd@163.com (T.C.-Y.L.)

**Keywords:** Raman spectroscopy, microvessels, blood glucose, non-invasive, PCA, BP-ANN, Clarke error grid

## Abstract

Raman spectra of human skin obtained by laser excitation have been used to non-invasively detect blood glucose. In previous reports, however, Raman spectra thus obtained were mainly derived from the epidermis and interstitial fluid as a result of the shallow penetration depth of lasers in skin. The physiological process by which glucose in microvessels penetrates into the interstitial fluid introduces a time delay, which inevitably introduces errors in transcutaneous measurements of blood glucose. We focused the laser directly on the microvessels in the superficial layer of the human nailfold, and acquired Raman spectra with multiple characteristic peaks of blood, which indicated that the spectra obtained predominantly originated from blood. Incorporating a multivariate approach combining principal component analysis (PCA) and back propagation artificial neural network (BP-ANN), we performed noninvasive blood glucose measurements on 12 randomly selected volunteers, respectively. The mean prediction performance of the 12 volunteers was obtained as an RMSEP of 0.45 mmol/L and R^2^ of 0.95. It was no time lag between the predicted blood glucose and the actual blood glucose in the oral glucose tolerance test (OGTT). We also applied the procedure to data from all 12 volunteers regarded as one set, and the total predicted performance was obtained with an RMSEP of 0.27 mmol/L and an R^2^ of 0.98, which is better than that of the individual model for each volunteer. This suggested that anatomical differences between volunteer fingernails do not reduce the prediction accuracy and 100% of the predicted glucose concentrations fall within Region A and B of the Clarke error grid, allowing acceptable predictions in a clinically relevant range. The Raman spectroscopy detection of blood glucose from microvessels is of great significance of non-invasive blood glucose detection of Raman spectroscopy. This innovative method may also facilitate non-invasive detection of other blood components.

## 1. Introduction

After cancer and cardiovascular disease, diabetes has recently become the third most common chronic disease, causing serious damage to human health [1]. Diabetes, which has been declared a global epidemic by the World Health Organization, is a worldwide epidemic affecting 422 million people and may pose a tremendous threat to public health in the coming years [2]. Diabetes is a chronic disease in which insulin cannot be produced or cannot be used properly by the body. Insulin needs to absorb glucose from the blood and generate energy. When the insulin cycle is defective, glucose will not be removed from the blood, causing an accumulation [3]. Leaving diabetes untreated may lead to severe consequences, including kidney failure, cardiovascular and birth defects [4]. Although a cure for diabetes has not been found, researches reveal that effective glycemic control reduces complications and prolongs life of diabetics for 5 to 8 years [5]. The most advanced glucose monitors, which use electrochemical testing strips, require diabetic patients to lance their fingers and test drops of blood several times a day [6]. This testing not only causes pain in diabetic patients several times a day, but also may pose an infection risk. The above reasons explain why blood glucose monitoring has not been carried out as often as recommended over the years [7,8,9]. Even with significant technical challenges, the goal of eliminating pain and increasing the convenience for diabetic patients has motivated scientists to develop non-invasive glucose monitoring devices [10]. For this reason, the creation of a non-invasive approach for blood glucose measurement has long been regarded as the holy grail. 

Optical methods, including microwave spectroscopy [11], optical coherence tomography [12,13], near-infrared (NIR) spectroscopy [14], polarimetry [15], Raman spectroscopy [16,17,18] and fluorescence techniques [19], have been considered as accurate and painless means of blood glucose detection. Among the distinct optical techniques used in glucose measurement, Raman spectroscopy is one of the most promising optical approaches [16,20]. Raman scattering offers molecular “fingerprinting” capability as a result of the inelastic interactions between the incident photons and molecular vibrations [21]. Raman spectroscopy has several advantages, including the following: without destruction to the sample, capability for qualitative measurements, excellent chemical stability, ability to obtain molecular structure information with high spatial resolution; and Raman spectroscopy does not require reagents or separation [16,22]. Compared to other optical methods, Raman spectroscopy has the unique superiority to provide clear and intelligent information for human skin, along with the glucose molecule [20,23,24]. Moreover, comparing with NIR absorption spectroscopy, Raman spectroscopy is less prone to incorporate opportunistic correlations by the calibration models [22].

Scientists continuously promote the development of non-invasive Raman blood glucose testing by detecting the various body fluids related to human blood glucose. The studies of using numerous body fluids including tear fluid [25,26], salivary [27], and sweat [28] instead of blood [29] have shown that Raman spectroscopy is a powerful tool for non-invasive detection of blood glucose. However, the problem is still to be resolved of not achieving high average correlation coefficient and longer time lag [30,31]. Tear, saliva and sweat have all been used as optical detection substance because they are easily accessible, related to blood glucose and completely non-invasive, and safe enough for human body. However, their shortcomings are also obvious, the glucose concentration is low, the pH variance is high, and the time lag is long. Especially the saliva, the remaining food or drinks will affect the accuracy of the data [8]. Several methods have been reported for the detection of blood glucose levels using in situ Raman spectroscopy. Since the depth of penetration of the excitation light is very shallow, it is only 200 μm, the stratum corneum (SC) and epidermis of most sites in the human body are so thick that the laser cannot reach the dermis while the microvessels only exist in the dermis [32]. Therefore, in the transcutaneous Raman blood glucose test, scientists usually only obtain the Raman spectrum of the SC and epidermis. That is to say, most of these spectra are derived from the ISF in the epidermis, not in the dermis.

Glucose in the microvascular of the dermis diffuses to the epidermis. A physiological lag is inevitable between blood and ISF glucose [29,33]. In addition, the concentration of ISF glucose is also significantly lower than that of blood glucose [34]. It is well known that the excitation efficiency of Raman scattering is very low, and its intensity is only one thousandth of Rayleigh scattering. In order to obtain a low concentration of ISF glucose percutaneously, many of the existing work has focused on improving the detection instrument to increase the Raman spectral intensity of blood glucose [35,36,37]. But the main challenge is that physiological lag creates an inconsistency in prediction model based on blood glucose concentrations and Raman spectra of ISF glucose in the epidermis [29]. These problems can be avoided if Raman information is obtained directly from the blood. Recently, Shao et al. have shown that by focusing the laser directly on selected blood vessels of live mice, strong background signals generated by surrounding tissues were reduced successfully [38]. Fortunately, the skin at the special anatomical site of the human body has an ultra-thin SC and a nearly transparent epidermis, while also having a high density of blood vessel, such as nailfold [39,40].

In this study, we collected the Raman scattering photons of blood by focusing excitation light on volunteer microvessels in the nailfold. The obtained Raman spectrum contains significant blood characteristic peaks. The blood glucose concentrations of the human body were predicted in combination with principal component analysis (PCA) and back propagation artificial neural network (BP-ANN). Compared to the results reported in the literature, the results from 12 volunteers showed that we obtained higher correlation coefficients and lower root mean square errors.

## 2. Results and Discussion

### 2.1. OCT Imaging of Fingertip and Nailfold

In this study, a portable spectral domain Optical Coherence Tomography (OCT) (MOPTIM, Shenzhen, Co., Ltd., Shenzhen, China) system was applied in collecting images with a light source whose central wavelength is 830 nm. The OCT was controlled by a portable personal computer system operation automatically, and it select a 2-D OCT image every minute. Before storage, the detector is demodulated by a lock-in amplifier and a low-pass filter in the software.

**Figure 1:**
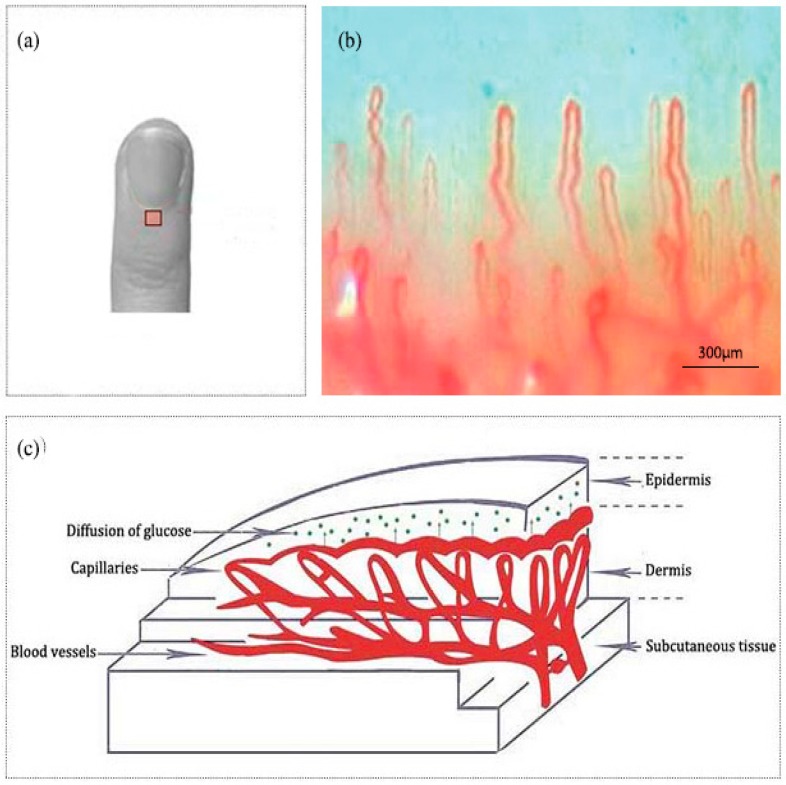
(**a**) A nailfold for the fourth finger including an area to be scanned. (**b**) The image of nailfold by the microcirculation detector. (**c**) Schematic of the nailfold skin with epidermis, dermis and subcutaneous tissue.

**Figure 2:**
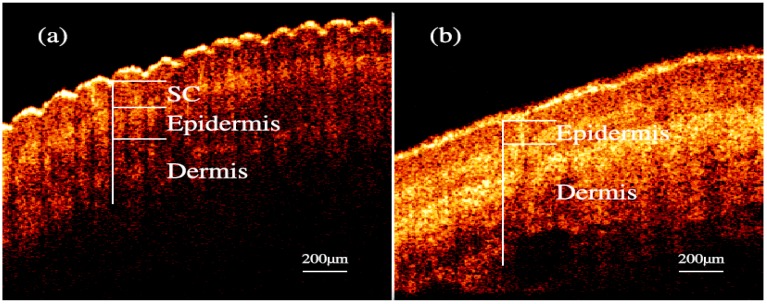
OCT images of skin. (**a**) fingertip. (**b**) nailfold.

Nailfold refers to the small area of the skin beneath the nail, which is shown in Figure 1a in red. Figure 1b shows the microscopic images of nailfold. The microcirculation detector and microcirculation analysis software of Beijing Defense Biological Technology Co., Ltd. (Beijing, China) were used. The microcirculation detector is a microscope with visible light illumination that is connected to a computer to store data which is usually used to observe the morphology of the microcirculation. The microvessels structure parallel to the skin surface of the nailfold can be seen clearly. This indicated that the nailfold’s SC and epidermis are very thin and they also have good light transmittance. Figure 1c shows the longitudinal section of the nailfold through human skin layers, including the SC, epidermis, dermis and subcutaneous tissue. The SC contains hornified cells and no ISF volume [41]. Epidermis is an avascular epithelial membrane and does not contain microvessels. The ISF volume in the epidermis increases from nearly absent in the SC to ~40% in the basal layers [41,42,43]. The dermis contains many arteries, venules and microvessels, including the vascular plexus that is interfacing dermis and subcutaneous tissue [29]. Figure 2a,b are the OCT images of fingertip and nailfold from a same volunteer, respectively. In Figure 2a, we can see that the stratum corneum at the fingertip is about 130 μm and the epidermis is about 180 μm, which means that the microvessels in the fingertip dermis are below 300 μm on the skin surface. Because the Raman spectroscopy is measured at the depth of 100–200 μm in the skin, this shows that the Raman spectra we gathered including much information from the stratum corneum and epidermis at the fingertips, and there is almost no information from the dermis [32]. In Figure 2b, the stratum corneum in the nailfold was not observed, which indicates that the stratum corneum here is thin enough to exceed the resolution of OCT. The nailfold contains a thinner epidermis that is only about 100 μm and we can gather more information from the dermis which the microvessels are only found in it.

### 2.2. Spectral Analysis

**Figure 3:**
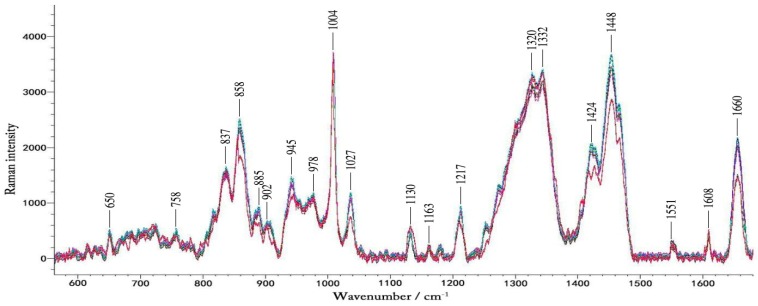
The typical Raman spectra of volunteers during the OGTT experiment. The different colors represent blood glucose levels at different times in one day.

Figure 3 shows typical Raman spectra of a volunteer in one-day OGTT experiment. We found that the changes in blood glucose concentration did not result in large changes in the Raman peaks visible to the naked eye. These spectra contain a lot of Raman peaks of the blood.

Table 1 summarizes spectra peaks of the microvessels and in vitro blood. The Raman spectra obtained by focusing laser on the microvessels in the nailfold. The Raman peaks appearing at 650 cm^−1^, 758 cm^−1^, 837 cm^−1^, 945 cm^−1^, 978 cm^−1^, 1004 cm^−1^, 1130 cm^−1^, 1163 cm^−1^,1217 cm^−1^, 1332 cm^−1^, 1551 cm^−1^ and 1660 cm^−1^ are obvious, which also exist in the blood [44,45]. This suggests that the Raman spectra of microvessels obtained at the nailfold is mainly from the blood in the microvessels. In the literature the Raman peaks of blood previously tested in the fingers and forearms, are not significant, and the spectra of the fingers and forearms are mainly from the epidermis and interstitial fluid [46,47]. During the lasting 2.5 h of the experiment, blood glucose was the only variable. The data of 30 pairs of Raman spectra and the corresponding blood glucose levels were gained in each glucose correlation test. It seemed that numerous spectral features changed over time, which were caused by changes in blood glucose. Changes in blood glucose concentration had effect on the intensity of the Raman peak. Because the Raman cross-section of glucose is small, in the Raman spectra detected by 12 volunteers, we did not find any Raman peaks changing regularly with changes in blood glucose concentration. That is, in the Raman spectra we detected from the microvessels, the glucose peak of the physiological concentration was not visible while other substances in the blood and tissues surrounding the blood vessels contribute more to these peaks. And it is difficult to analysis the spectra based on the single peak of the lower concentration compounds like glucose [16]. Therefore, a more thorough data analysis method was then implemented.

### 2.3. PCA and BP-ANN Model

Considering changes in blood glucose can also cause changes in tissue turbidity and light transmittance, and blood glucose may interact with other substances in the blood to cause changes in other substances, which may cause nonlinearities in blood glucose concentration and Raman peaks [16]. In this paper, we used the BP-ANN as a nonlinear algorithm which can handle both nonlinear and linear relationships for increasing the testability of the low blood glucose concentration from Raman spectra. During the data processing, PCA and BP-ANN were used together to predict the blood glucose concentrations from the non-invasive measurements.

In order to eliminate redundant interference, PCA was used for reducing the dimensions of the spectral matrix [49]. Then the optimal principal component (PC) could be selected as the inputs of BP-ANN. It is necessary to compress data for high-dimensional data sets containing multiple large variables. PCA is considered as a data compression technique for spectroscopic data. The common characteristics of the Raman spectra obtained in the process of glucose variations from low to high values can be analyzed by PCA. Extracting the main components of the Raman spectra of volunteer 1, the contribution rates of the first three principal components, PC1/PC2 and PC3, were 80.065%, 14.982%, 3.157%, and the cumulative contribution rate was 98.204%. These data can explain most of the spectral feature differences. The PC of other volunteers also has a high proportion. Therefore, we replace the original spectral data with the PCA processed data as the input value of the BP-ANN.

After data pretreatment by PCA, we used the BP-ANN model for predicting the blood glucose. Over the last few years, ANN has been used more and more popular in the qualitative and quantitative analysis because of its advantages including anti-interference, anti-noise and strong nonlinear transmission capability. Among them, the most widely used is the BP-ANN model [50]. With the ability to realize highly nonlinear mapping between input and output, the BP-ANN model is a formidably research system which is demonstrated that the model can achieve any continuous nonlinear curve [51]. The BP-ANN is chosen for the reason that it can handle both linear and nonlinear relationships. A BP-ANN is usually composed of three layers: input layer, hidden layer and output layer. The tansig function is widely used in the transfer function between the input layer and the hidden layer, and the purelin function is used between the hidden layer and the output layer. The input layer has the same number of input nodes as the number of principal components. We could improve the network performance and create an ideal calibration model by changing the number of hidden layer nodes. The output layer had one neuron, the glucose concentration. The training time was set to 18 times.

Based on the studies, we have obtained the optimal network structure, in which the number of nodes of the input layer, hidden layer and output layer are 3, 4 and 1. After the model parameters are selected, the preferred parameters of the BP-ANN model are as follows: the number of neurons is 3, the number of learning is 100, the learning rate is 0.01, the learning momentum is 0.7, and the learning goal is 0.001. Through the model training, the BP-ANN model training error meets the accuracy requirements, and the model grasps the information in the sample.

### 2.4. Model Reliability

The two correction parameters: the root mean square error of prediction (RMSEP) (1) and the squared correlation coefficient (R^2^) (2) are usual metrics that evaluate the stability and the reliability of the dependability model:

(1) $$\mathrm{RMSEP}=\sqrt{\frac{\sum_{i=1}^{n}{\left(ŷi-yi\right)}^{2}}{n}}$$

(2) $$R^{2}=1-\frac{\sum_{i=1}^{n}{\left(yi-ŷi\right)}^{2}}{\sum_{i=1}^{n}{\left(yi-\bar{y}\right)}^{2}}$$

where *yi* is the result of the reference measurement, *n* is the number of samples included in the forecasting set, *ŷi* is the estimated result for sample *i* and $\bar{y}$ is the average of all tested values.

### 2.5. Statistical Analysis

**Figure 4:**
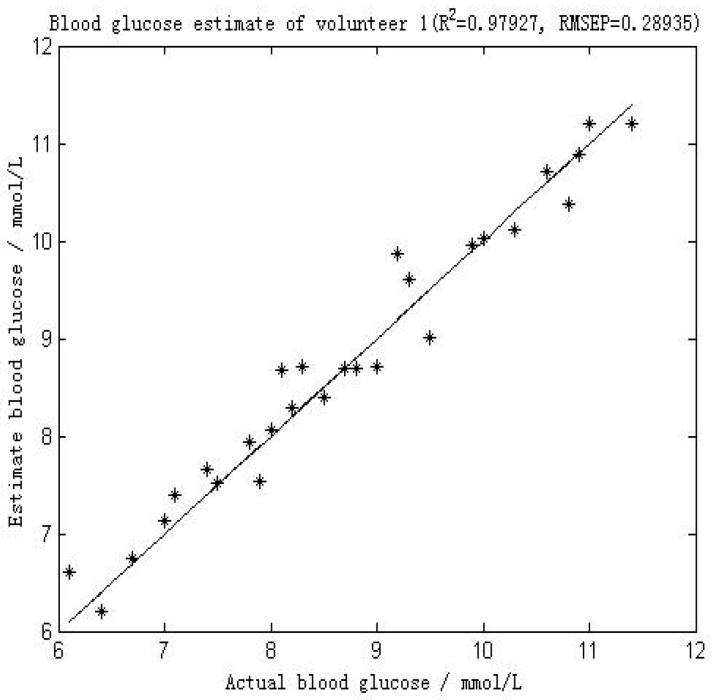
Results of the blood glucose estimation of volunteer 1 using a PCA and BP-ANN model in which part of Raman spectroscopy region was used (552–1675 cm^−1^). R^2^ was 0.97927 and the RMSEP was 0.28935 mmol/L.

**Figure 5:**
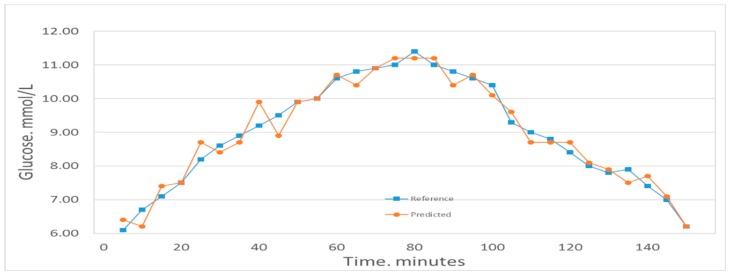
Predicted glucose concentrations tracking the reference values for one volunteer.

Figure 4 shows the comparison between the first volunteer’s real blood glucose and his estimated blood glucose on Day 10. The calculated RMSEP is 0.28935 mmol/L, with R^2^ of 0.97927. The relatively low RMSEP indicates that the model has a high forecasting capability and therefore that this calibration is precise. The value of R^2^ indicates that there appears an excellent linear relationship between the real value and the predicted validated results. Figure 5 shows a comparison of the predicted glucose concentration to the corresponding reference data from the first volunteer. During rapid blood glucose changes, the lag time of the microvessels in the nailfold of the volunteer was almost 0 min. This is mainly because the spectra were collected from volunteer’s blood of nailfold where we can focus on the microvessels. That procedure was applied to the 12 volunteers. The result of the validated calibrations for the data set is summarized and shown in Table 2.

The mean prediction performance of the 12 volunteers was obtained as RMSEP of 0.45 mmol/L and R^2^ of 0.95. The Day 1–Day 9 of the 12 volunteers were used as the calibration set, and the Day 10 was used as the prediction set, we obtained the total predicted performance of all volunteers with RMSEP of 0.27 mmol/L and R^2^ of 0.98. We observed that the method performs outstandingly for the calibrations for all volunteers. The calibration spectra drayed from the microvessels in the nailfold in vivo measurement shows high quality, providing clear evidence of the ability to sense the blood glucose immediately.

**Figure 6:**
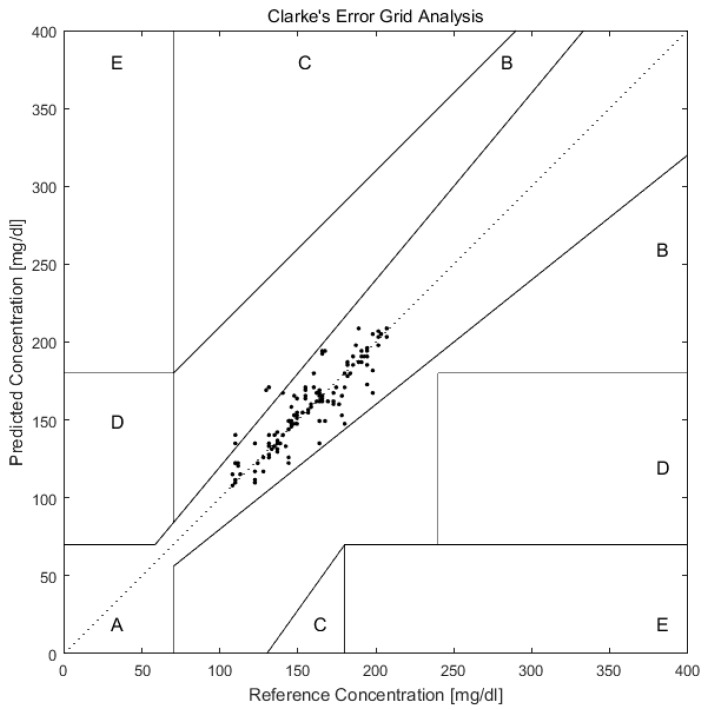
Clarke error grid plot of glucose concentration predictions in microvessels versus actual blood glucose concentration of 12 volunteers in Day 10. The predictions are all in the A and B regions, which are the desirable regions for clinical accuracy.

As shown in Figure 6, after predicting the blood glucose of Day 10 of all 12 volunteers with PCA and BP-ANN, we plotted the predicted and reference values in the Clarke error grid. The Clarke error grid was established by Clarke and co-workers to evaluate the clinical utility of systems for blood glucose monitoring [52,53,54]. Now it is usually used to assess the accuracy of blood glucose measurements to the standard reference value. It is divided into five regions. Region A and Region B represent valuable correct clinical decision with Raman predictions and acceptable clinical error in either direction. While the Region C, D, E are increasingly harmful incorrect decisions [55]. From the Figure 6, we can see that all prediction values exist on Region A and Region B. This can further support the applicability of transcutaneous spectral measurements in microvessels for glucose monitoring.

Its enhanced sensitivity and reliability will meet medical use standards [8], and the lag time in microvessels of the volunteer was almost 0 min. This site effectively improves the accuracy of non-invasive blood glucose detection and greatly exceeds the precision of previous test results using Raman spectroscopy. This kind of non-invasive technique will be valuable for a wide variety of laboratory tests and clinical settings. The nailfold’s predictiveness of blood glucose is superior to the previous predictions of the forearm (MAE = 7.8%, R^2^ = 0.83) and fingertip (RMSEP = 13.63 mg/dL, R^2^ = 0.91), which is an improvement of non-invasive blood glucose detection using Raman spectroscopy [46,56].

## 3. Materials and Methods

### 3.1. Experimental Setup

**Figure 7:**
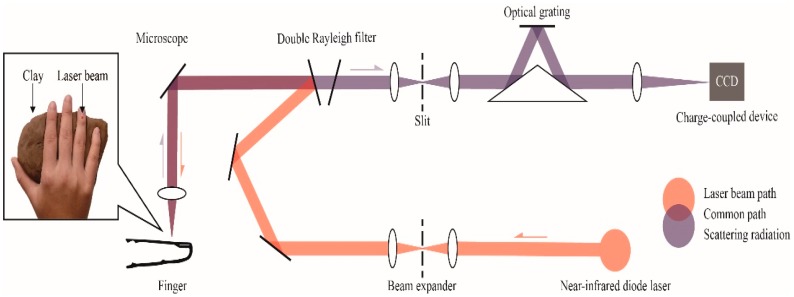
Raman spectroscopic apparatus for noninvasive glucose measurement. An inset shows the detection site located in the right hand of the volunteer, volunteers’ hands are placed on clay to reduce motion artifacts.

A Renishaw inVia confocal Raman spectrometer (Renishaw, Inc., New Mills, UK) was applied in collecting all data from volunteers. A block diagram of the Raman measurement apparatus is presented in Figure 7. The solid box in the inset in Figure 7 is the detection site located in the right hand of the volunteer; the laser was focused on the microvessels in the nailfold. Raman spectra were obtained using the micro-Raman system. It is equipped with a 300 mW near-infrared diode laser with a wavelength of 785 nm excitation. The laser beam was positioned with a Raman imaging microscope (Leica, Shanghai, China) equipped with a 20× objective lens (numerical aperture = 0.35). A charge-coupled device (CCD) array detector was used to detect signals from a 1200 grooves/mm grating light path controlled by Renishaw WiRE software version 3.2. Before the experiment, a silicon wafer was used to calibrate the Raman system. Each spectrum was collected by using 6 accumulations of 4 s exposure time within the range 552–1675 cm^−1^. The incident power on the samples was around 13 mW, which is well below the maximum permissible exposure for continuous wave laser skin illumination at 785 nm. None of the volunteers suffered any discomfort during the test or appeared any skin injury afterwards.

### 3.2. Study Subjects

The experiments were implemented out on 12 volunteers (six men and six women) who were 23 years old or older (average age 25 years). The subjects were in excellent health and taking no medications. Signed informed consents were obtained from all participants prior to experiment. Non-invasive blood glucose measurement was carried out according to the following experimental protocol. Healthy volunteers were seated and allowed to acclimate for 30 min at room temperature (25 °C) prior to measurement.

For the purpose of having a wide variety of blood glucose concentrations in the non-invasive blood glucose measurement experiment, OGTT was recommended; the OGTT is a test measuring the response to a glucose load for the clinical diagnosis of diabetes [48,57]. For a healthy person, various glucose concentrations can be obtained within several hours during this test. The OGTT is a commendable experimental approach for obtaining a calibration model with a certain concentration variety, which provides another way to study non-invasive blood glucose measurement by Raman spectroscopy. The detection site was cleaned with medical-grade alcohol prior to each experiment, in order to eliminate the influence of dirt or sweat.

### 3.3. Experimental Protocol

The experiment was usually performed in the morning, when after 12 h of fasting, the volunteers were asked to drink 250 mL water with 75 g glucose in 5 min. The Raman spectrum was gathered from the nailfold of the volunteer’s fourth finger. The fingertip was placed under the probe head, and the objective lens was adjusted to focus on the microvessels of each volunteer’s nailfold. Clay was used to stabilize the fingers, and humidity between the clay and the surface of the fingers can fix the nailfolds on the light spot. With the help of clay, slightly uncontrolled shaking and manual displacement can be eliminated, and the stability of human spectra will be improved. The volunteers were in a comfortable state, and the wrists, tested finger and arm were well fixed. Every spectrum was formed by averaging six consecutive 4 s acquisitions. Spectra were got every 5 min throughout 2.5 h, making up a ‘‘measurement series’’ for each volunteer (30 spectra per series).

During the period, the volunteers were asked not to move to minimize motion artifacts, and no food and drinks were allowed. In addition, the measurement site, measurement pressure and psychological state of the volunteers were kept constant as much as possible during the sampling. The fingers of the unengaged hand were pricked every 5 min by a glucose meter, the OneTouch (Johnson & Johnson, New Brunswick, NJ, USA), to obtain reference blood glucose measurements. During the measurement, the blood glucose concentration usually doubles and then returned to the initial value. For all volunteers, the blood glucose concentrations ranged from 5.8 to 12.0 mmol/L.

### 3.4. Data Treatment

Data processing and advanced statistical analysis were implemented using MATLAB (R2014a) and the software R (2.8.1) (Auckland, New Zealand). Each spectrum was first treated with WiRE 3.2 software to eliminate cosmic ray interference, and then was preprocessed by a fifth polynomial fit subtraction method to baseline correction with the software R. Before a BP-ANN model was built for prediction of blood glucose level, PCA carried out to reduce the dimensions of input variables.

According to the researches of PCA, BP-ANN model was developed by choosing tansig as the transfer function of the hidden layer. In the BP-ANN algorithm, personal spectral data is procedurally divided into two subsets: the first one is called a calibration set and the other is a prediction set. The calibration data are used to establish a calibration vector that is used to evaluate the accuracy of the calibration model.

The performance of the model could be evaluated with the comparison of the differences between the concentration predicted by the model and the concentration measured by the reference method. In the BP-ANN algorithm, 300 samples for each volunteer were split into a model set, and a predicting set at random; the model set included 270 samples, and the latter set contained 30 samples. In the BP-ANN algorithm, the data from the Day 1 to Day 9 were collected to build a model set, which was used to predict the blood glucose levels of the Day 10.

## 4. Conclusions

In our study, Raman spectra containing significant blood characteristic peaks were obtained by focusing the laser on the microvessels in the superficial layer of the nailfold. This shows that although the transmission depth of the Raman spectroscopy in the skin is about 200 μm, for most sites of the human body, we can only collect the Raman signal of the SC and the epidermis, and it is impossible to obtain the blood which only presents in the dermis. However, there are some specific sites in the human body which allow this. Because of the extremely thin SC and the high light transmittance of the epidermal layer, it is possible to obtain the Raman spectra mainly originating from blood in the dermis. Unlike the previously reported transdermal Raman spectroscopy method for detecting blood glucose, the Raman spectra we used mainly derived from blood replaced the Raman spectra derived from the epidermis and interstitial fluid. We used the algorithm combining PCA and BP-ANN to predict the blood glucose for 12 volunteers. The mean prediction performance of the 12 volunteers was obtained as an RMSEP of 0.45 mmol/L and R^2^ of 0.95. And the total predicted performance of all volunteers was obtained with an RMSEP of 0.27 mmol/L and R^2^ of 0.98. Using the Clarke error grid to evaluate the method, we got good results. 100% of the predicted glucose concentrations fall within Region A and B, making acceptable predictions in a clinically relevant range. The predictiveness is superior to the previous predictions in literature. The reason for achieving such high prediction accuracy may be that the Raman spectra we used mainly derived from blood instead of the epidermis and interstitial fluid, thus avoiding the physiological lag between blood glucose and ISF glucose, and ensured consistency with the used spectra in the calibration model. The non-invasive blood glucose monitoring of Raman spectroscopy obtained from the microvessels blood may indicate the direction for the future. Furthermore, this novel method of obtaining Raman spectra of blood by focusing the laser on microvessels in the skin can also be applied to rapid non-destructive detection of other blood components, such as prediction of hemoglobin levels and measurement of glycated hemoglobin.

## Figures and Tables

**Table 1:** Assignments of Raman peaks that are identified in the spectra of the microvessels and blood [45,46,47,48].

Peak Position (cm^−^^1^)		Assignments	Components
Microvessels	Blood		
650	643	p:C–S str	Ascorbic acid
758	752	ν_15_	Trp
837	827	γ10	Fructose
858	855	ν(C–C)	Tyr, lac
885	-	-	-
902	898	p:C-C skeletal	Tyr
945	940	ν(C–C)	Citric acid
978	971	p: Skeletal vibr	Fibrin
1004	1004	ν-ring	Phe
1027	1026	δ(=C_b_H_2_)asym	Lac
1130	1129	ν_5_,	Lac
1163	1157	ν_44_	Heme
1217	1212	ν_5_ + ν_18_	Heme
1320	1321	p:CH2 twist	Try
1332	1341	ν_41_	Trp
1424	1423	ν_28_	Acetates
1448	1450	δ(CH_2_/CH_3_)	Trp
1551	1546	ν_11_	Heme
1608	1603	ν (C=C)_venyl_	Heme
1660	1653	Amide I	Heme

Abbreviations: ν & δ: In-plane modes, γ: Out -of- plane modes, asym: asymmetric, Str: stretching, p: protein.

**Table 2:** RMSEP and R^2^ for the 12 volunteers.

Volunteer	RMSEP (mmol/L)	R^2^
1	0.28935	0.97927
2	0.38727	0.95650
3	0.39516	0.95986
4	0.50273	0.93288
5	0.48272	0.93581
6	0.46750	0.94744
7	0.38724	0.96071
8	0.79781	0.87743
9	0.39834	0.95375
10	0.36541	0.96559
11	0.48413	0.94198
12	0.48610	0.93737
Mean	0.45365	0.94572
All	0.26601	0.98392
